# Supplementary material for: Escape mutations circumvent a tradeoff between resistance to a beta-lactam and resistance to a beta-lactamase inhibitor
Source: Nat Commun. 2020 Apr 24;11:2029. doi: 10.1038/s41467-020-15666-2 (PMC7181632; doi:10.1038/s41467-020-15666-2)
Supplement: Supplementary file 1 — Supplementary Information [file 41467_2020_15666_MOESM1_ESM.pdf]

Supplementary information

Escape mutations circumvent a tradeoff between resistance to a beta-lactam and resistance to a beta-lactamase inhibitor

Russ et al.

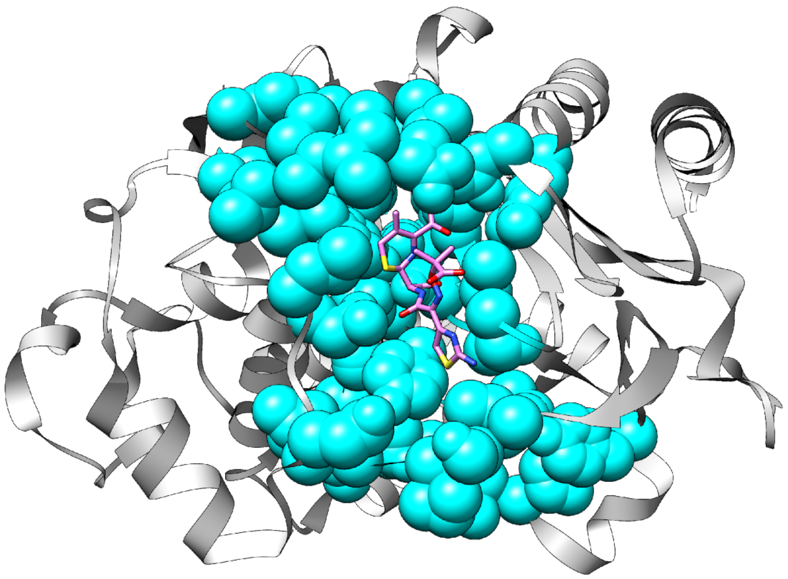

Supplementary Figure 1. AmpC pocket. Protein is shown in grey ribbon, residues in the pocket of AmpC are shown in cyan spheres, while the beta-lactam antibiotic ceftazidime is shown in sticks and colored by atom (C atoms colored pink, N blue, O red and S yellow).

a

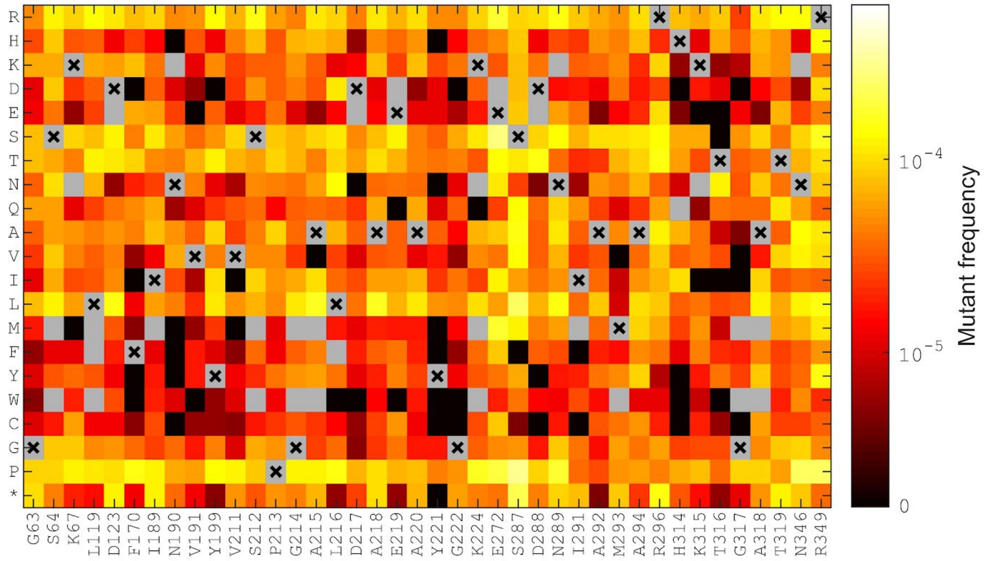

b

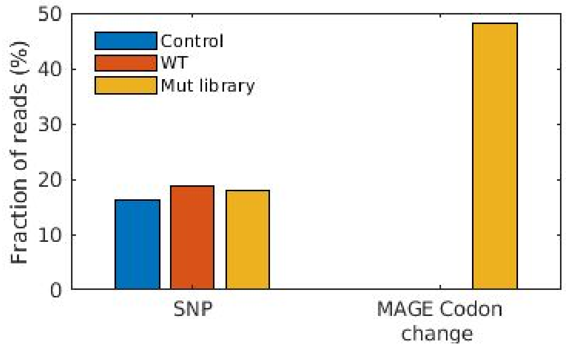

Supplementary Figure 2. Mutants abundance in the mutants library. a. Mutants frequency in the mutant library as identified by high-throughput sequencing of regions in the *ampC* gene. Since the mutated codons in the library are at least two nucleotides away from the original codon, not all amino acid substitutions are accessible. Non-accessible substitutions are marked in gray and the original amino acid with an X. The frequency data presents 40 of the 44 mutated residues excluding positions 148, 150, 151, and 152 which lie in the middle of the amplicon and were not well sequenced. Overall, the mean frequency of mutants is 5.3×10^{-3} % ± 4.2×10^{-3} % of the reads. **b.** Reads that contain one SNP (a single nucleotide change compared to the wild type sequence) as well as reads that contain mutations that resembles the designed MAGE mutations were identified from high-throughput sequencing of bases 768-999 in the *ampC* gene. The two types of mutations were identified in

WT *ampC* strain and in the MAGE mutant library. Both strains were selected on a high drug concentration. In addition, mutations were identified in a control strain that was not selected on antibiotics (Methods). While all strains show similar frequency of SNPs, MAGE mutants appear mainly in the mutant library (48%), while very rare in the WT libraries (0.3%) and not at all in the control library.

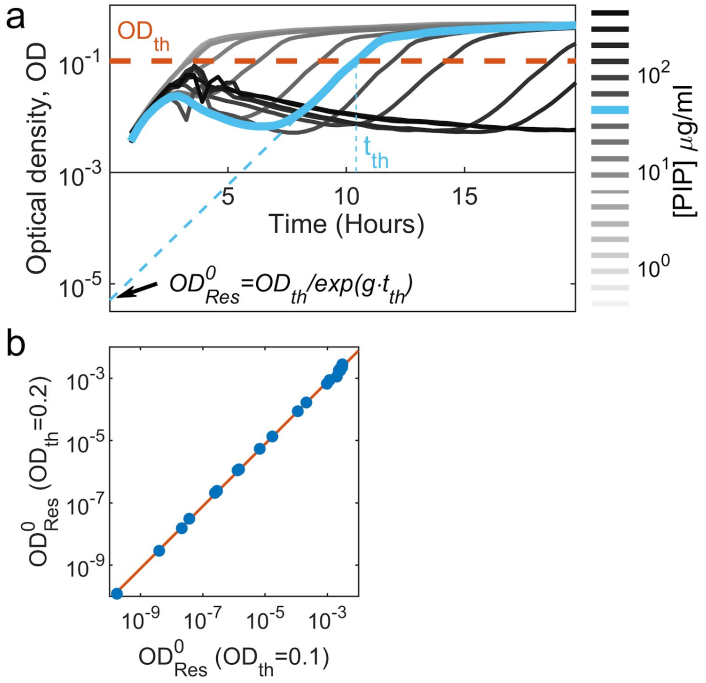

Supplementary Figure 3. Calculating the initial number of viable mutants from growth measurements of the mutant library. a. Growth of the mutant library exposed to increased concentration of PIP (black to light gray lines). To calculate the initial density of viable bacteria OD^0_{Res} we extrapolate the exponential growth phase of the culture to calculate its OD at time 0 (dashed cyan line). Since bacterial density during exponential growth in rate g is $OD_{(t)} = OD_0 \cdot exp(g \cdot t)$ the initial density of viable cells is $OD^0_{Res} = OD_{th}/exp(g \cdot t_{th})$. **b.** The initial density of viable mutants, OD^0_{Res}, calculated from the time, t_{th}, required to cross different density thresholds, OD_{th}, for mutant library growing on a range of piperacillin concentrations. The initial densities calculated for the different OD_{th} were highly correlated (fitted reggression line with slope of 1 in red) implies that our results do not depend on this arbitrary threshold .

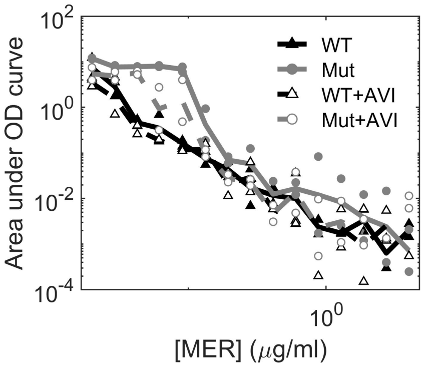

Supplementary Figure 4. *ampC* overexpression does not increase bacterial resistance to meropenem. Bacterial growth of the mutant library and the WT strain, as estimated by the area under the OD curve,, under stress of increasing MER concentrations with and without AVI. Growth of all strains was similar, implying that AmpC does not confer resistance to MER even when mutated.

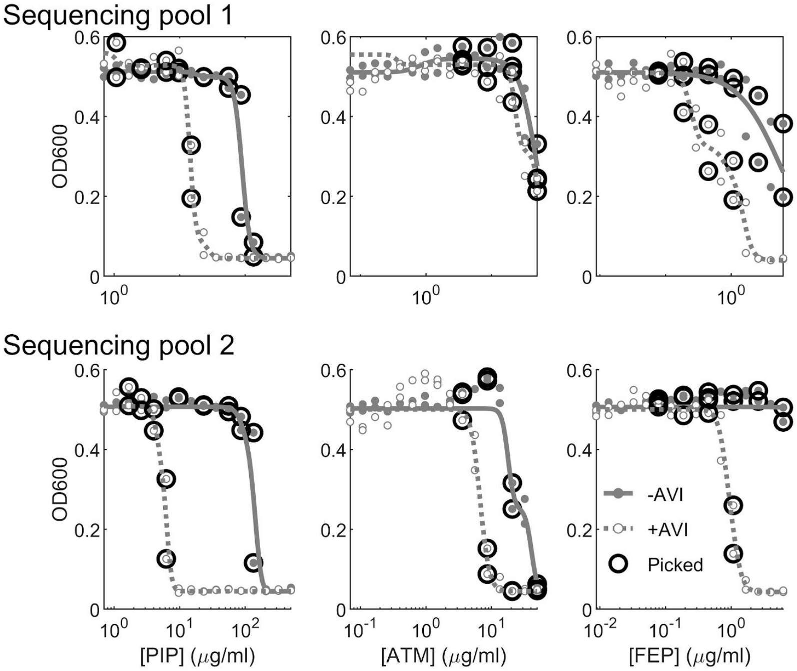

Supplementary Figure 5. A subset of mutant libraries cultures selected on gradients of antibiotics with and without avibactam was picked for high-throughput sequencing. Mutant libraries comprised of mutants with substitutions in the region of sequencing pool 1 (top, nucleotides 141-624) and sequencing pool 2 (bottom, nucleotides 768-999) were cultured in LB on gradients of three different drugs with and without avibactam. A subset of the cultures (circles) was picked for genetic analysis.

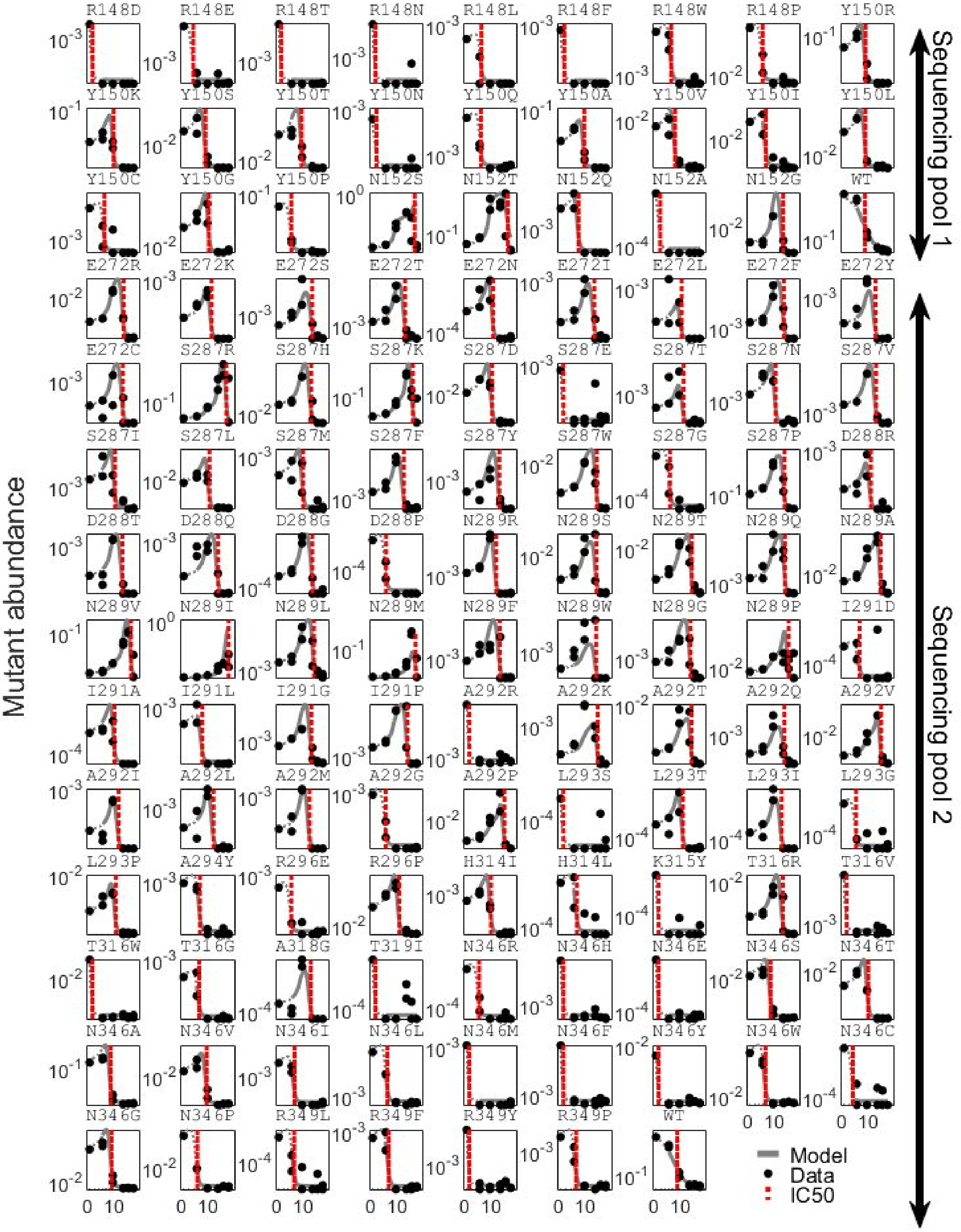

Mutant abundance
[PIP] (μg/ml)
Sequencing pool 1
Sequencing pool 2
Model
Data
IC50
R148D R148E R148T R148N R148L R148F R148W R148P Y150R
Y150K Y150S Y150T Y150N Y150Q Y150A Y150V Y150I Y150L
Y150C Y150G Y150P N152S N152T N152Q N152A N152G WT
E272R E272K E272S E272T E272N E272I E272L E272F E272Y
E272C S287R S287H S287K S287D S287E S287T S287N S287V
S287I S287L S287M S287F S287Y S287W S287G S287P D288R
D288T D288Q D288G D288P N289R N289S N289T N289Q N289A
N289V N289I N289L N289M N289F N289W N289G N289P I291D
I291A I291L I291G I291P A292R A292K A292T A292Q A292V
A292I A292L A292M A292G A292P L293S L293T L293I L293G
L293P A294Y R296E R296P H314I H314L K315Y T316R T316V
T316W T316G A318G T319I N346R N346H N346E N346S N346T
N346A N346V N346I N346L N346M N346F N346Y N346W N346C
N346G N346P R349L R349F R349Y R349P WT

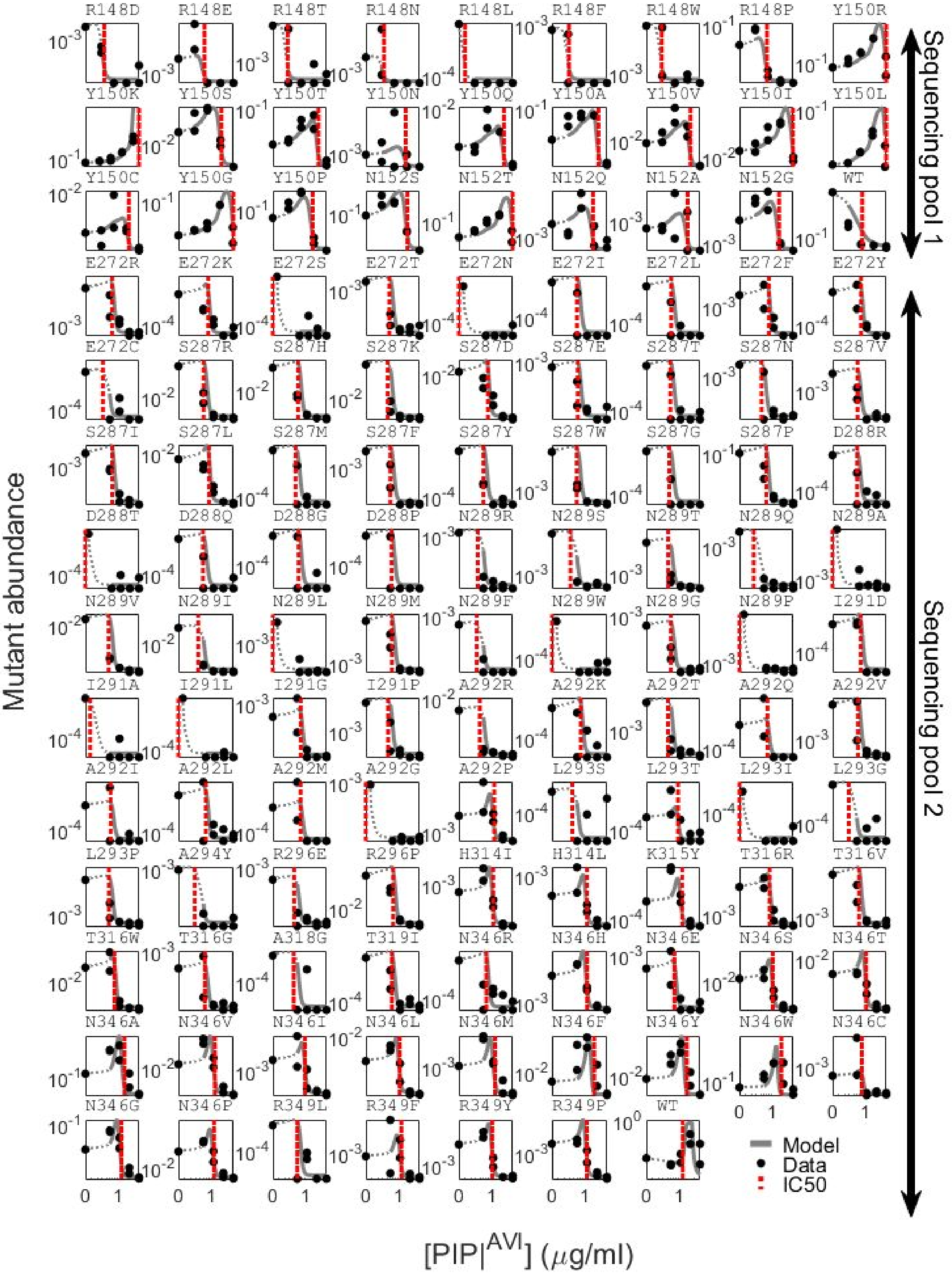

Mutant abundance
[PIP|AVI] (μg/ml)
Sequencing pool 1
Sequencing pool 2
Model
Data
IC50
R148D R148E R148T R148N R148L R148F R148W R148P Y150R
Y150K Y150S Y150T Y150N Y150Q Y150A Y150V Y150I Y150L
Y150C Y150G Y150P N152S N152T N152Q N152A N152G WT
E272R E272K E272S E272T E272N E272I E272L E272F E272Y
E272C S287R S287H S287K S287D S287E S287T S287N S287V
S287I S287L S287M S287F S287Y S287W S287G S287P D288R
D288T D288Q D288G D288P N289R N289S N289T N289Q N289A
N289V N289I N289L N289M N289F N289W N289G N289P I291D
I291A I291L I291G I291P A292R A292K A292T A292Q A292V
A292I A292L A292M A292G A292P L293S L293T L293I L293G
L293P A294Y R296E R296P H314I H314L K315Y T316R T316V
T316W T316G A318G T319I N346R N346H N346E N346S N346T
N346A N346V N346I N346L N346M N346F N346Y N346W N346C
N346G N346P R349L R349F R349Y R349P WT

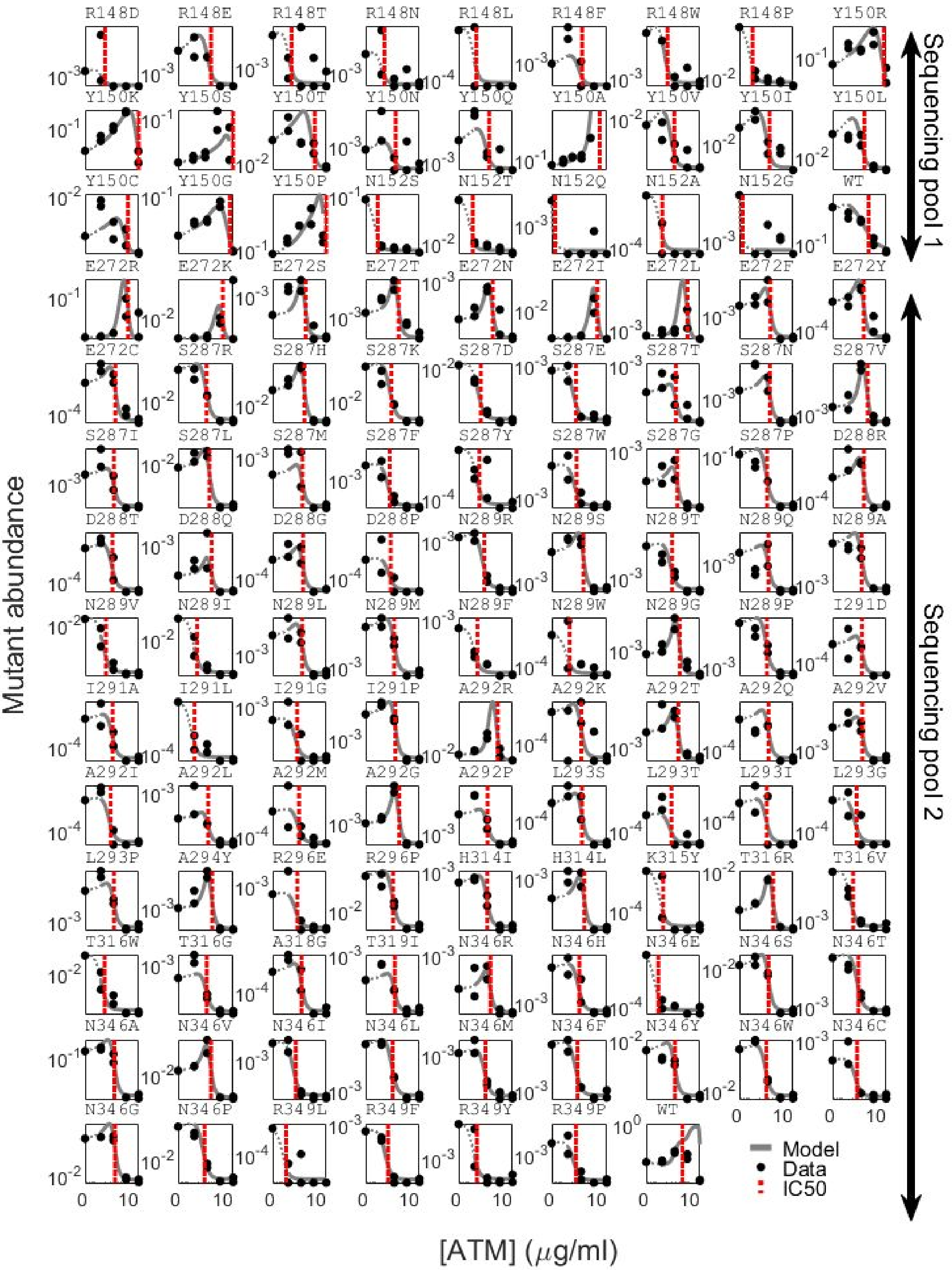

Mutant abundance
[ATM] (μg/ml)
Sequencing pool 1
Sequencing pool 2
Model
Data
IC50
R148D R148E R148T R148N R148L R148F R148W R148P Y150R
Y150K Y150S Y150T Y150N Y150Q Y150A Y150V Y150I Y150L
Y150C Y150G Y150P N152S N152T N152Q N152A N152G WT
E272R E272K E272S E272T E272N E272I E272L E272F E272Y
E272C S287R S287H S287K S287D S287E S287T S287N S287V
S287I S287L S287M S287F S287Y S287W S287G S287P D288R
D288T D288Q D288G D288P N289R N289S N289T N289Q N289A
N289V N289I N289L N289M N289F N289W N289G N289P I291D
I291A I291L I291G I291P A292R A292K A292T A292Q A292V
A292I A292L A292M A292G A292P L293S L293T L293I L293G
L293P A294Y R296E R296P H314I H314L K315Y T316R T316V
T316W T316G A318G T319I N346R N346H N346E N346S N346T
N346A N346V N346I N346L N346M N346F N346Y N346W N346C
N346G N346P R349L R349F R349Y R349P WT

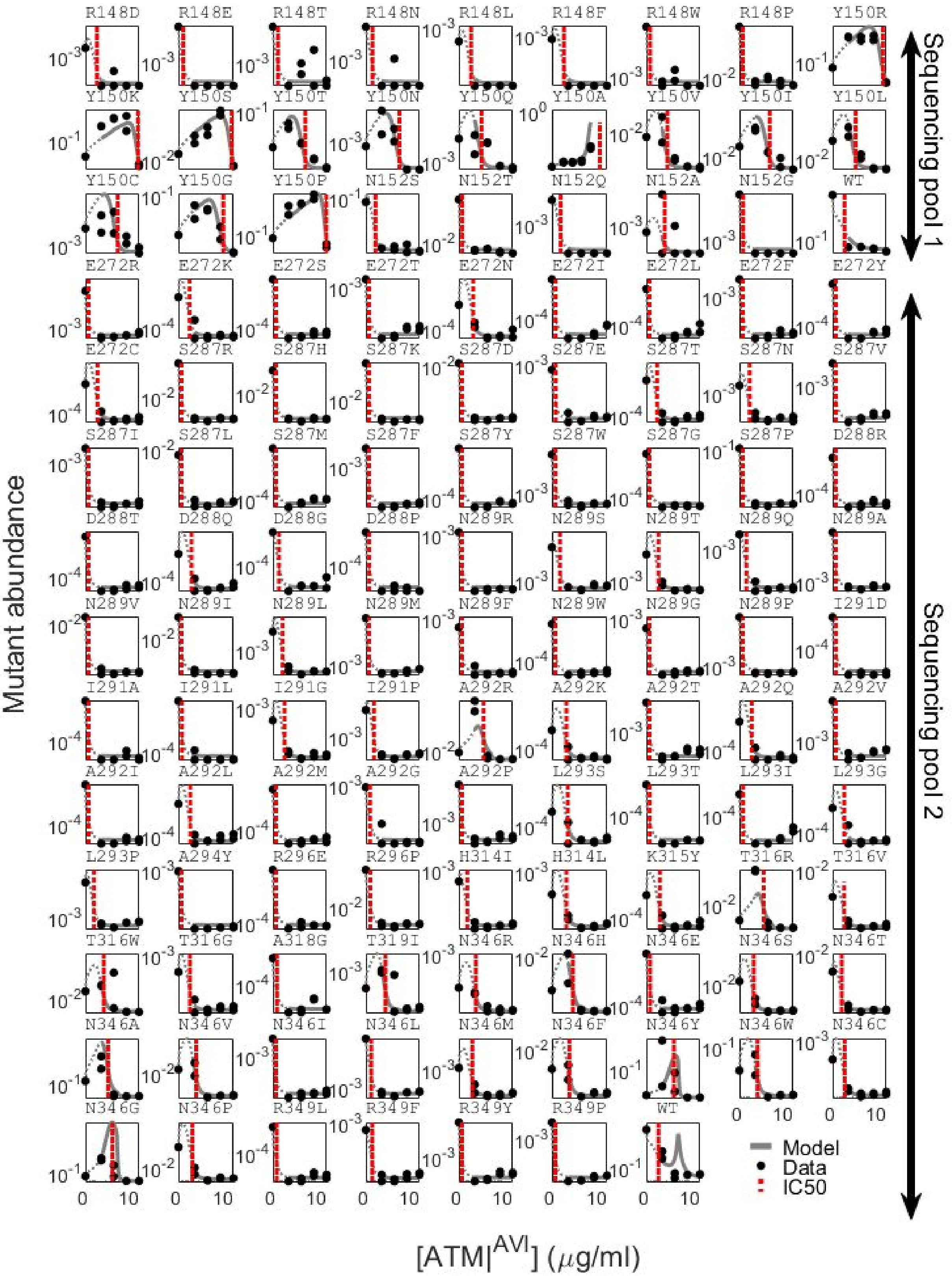

Mutant abundance
[ATM]^AVI] (μg/ml)
Sequencing pool 1
Sequencing pool 2
Model
Data
IC50

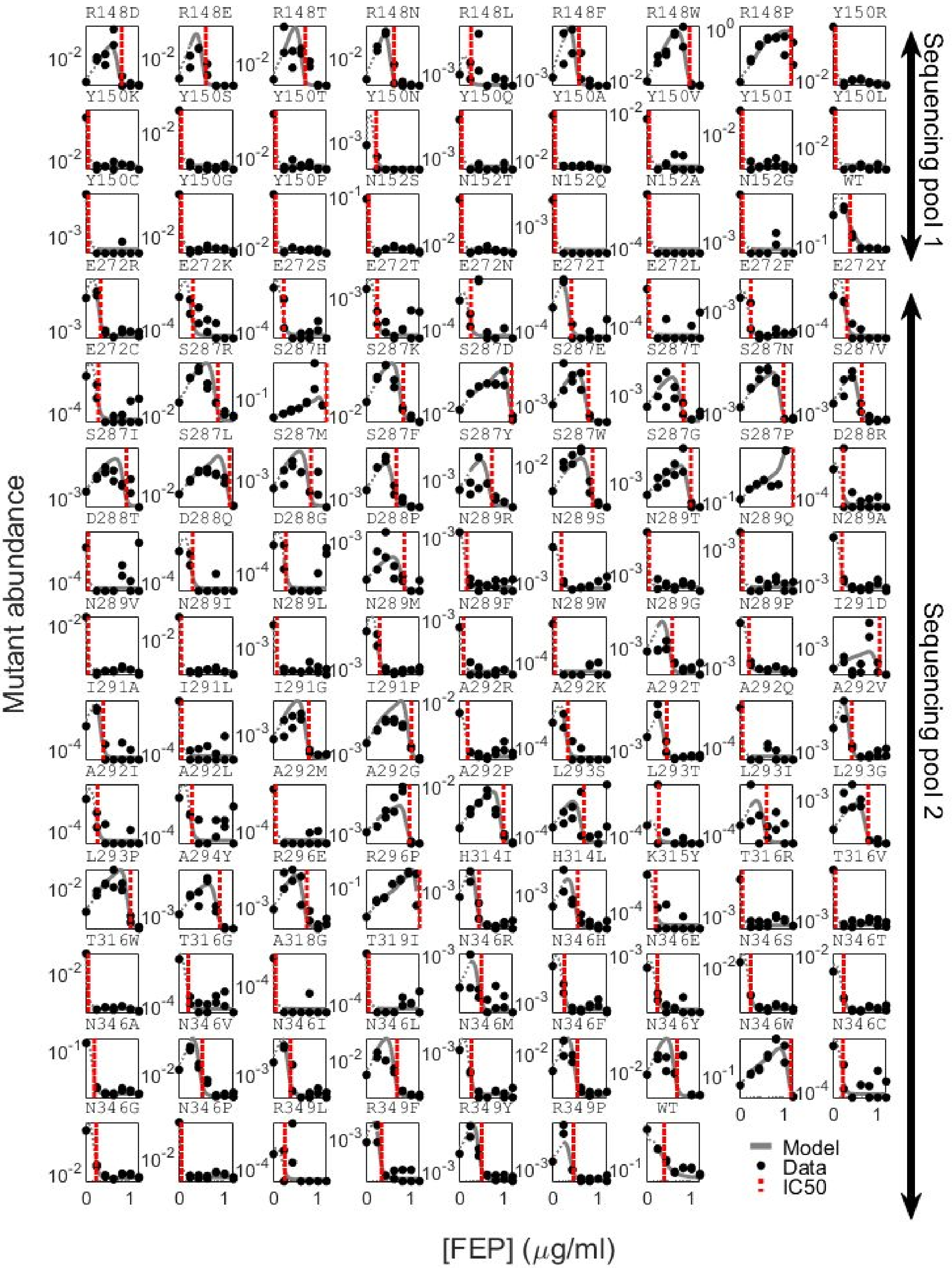
R148D R148E R148T R148N R148L R148F R148W R148P Y150R
Y150K Y150S Y150T Y150N Y150Q Y150A Y150V Y150I Y150L
Y150C Y150G Y150P N152S N152T N152Q N152A N152G WT
E272R E272K E272S E272T E272N E272I E272L E272F E272Y
E272C S287R S287H S287K S287D S287E S287T S287N S287V
S287I S287L S287M S287F S287Y S287W S287G S287P D288R
D288T D288Q D288G D288P N289R N289S N289T N289Q N289A
N289V N289I N289L N289M N289F N289W N289G N289P I291D
I291A I291L I291G I291P A292R A292K A292T A292Q A292V
A292I A292L A292M A292G A292P L293S L293T L293I L293G
L293P A294Y R296E R296F H314I H314L K315Y T316R T316V
T316W T316G A318G T319I N346R N346H N346E N346S N346T
N346A N346V N346I N346L N346M N346F N346Y N346W N346C
N346G N346P R349L R349F R349Y R349P WT
Model
Data
IC50
Mutant abundance
[FEP] (μg/ml)
Sequencing pool 1
Sequencing pool 2

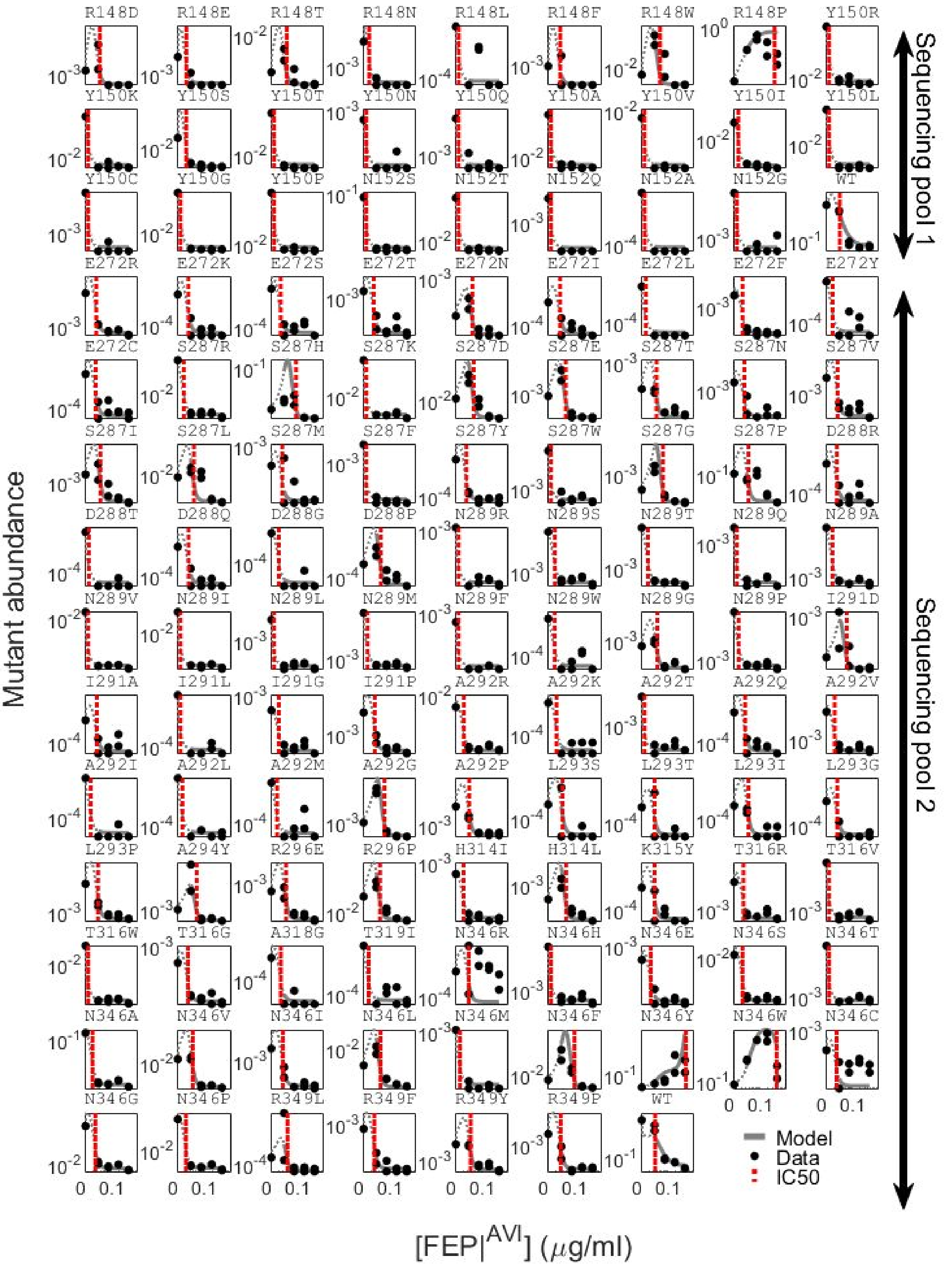

R148D R148E R148T R148N R148L R148F R148W R148P Y150R
Y150K Y150S Y150T Y150N Y150Q Y150A Y150V Y150I Y150L
Y150C Y150G Y150P N152S N152T N152Q N152A N152G WT
E272R E272K E272S E272T E272N E272I E272L E272F E272Y
E272C S287R S287H S287K S287D S287E S287T S287N S287V
S287I S287L S287M S287F S287Y S287W S287G S287P D288R
D288T D288Q D288G D288P N289R N289S N289T N289Q N289A
N289V N289I N289L N289M N289F N289W N289G N289P I291D
I291A I291L I291G I291P A292R A292K A292T A292Q A292V
A292I A292L A292M A292G A292P L293S L293T L293I L293G
L293P A294Y R296E R296P H314I H314L K315Y T316R T316V
T316W T316G A318G T319I N346R N346H N346E N346S N346T
N346A N346V N346I N346L N346M N346F N346Y N346W N346C
N346G N346P R349L R349F R349Y R349P WT
Mutant abundance
[FEP|AVI] (μg/ml)
0
0.1
Model
Data
IC50
Sequencing pool 1
Sequencing pool 2

Supplementary Figure 6. Mutants susceptibility to different drugs is estimated by fitting reads abundance to a mathematical model of inter-mutant competition. The relative abundance (RA_{Mut}) of each mutant is estimated from high-throughput sequencing and used to calculate the 'mutant abundance', $RA_{Mut}\ x\ OD_{Culture}$ (Duplicate measurements, black dots). The abundance data were fitted to a dose-response model to calculate the mutant resistance ($IC50$, red dashed line) based on inter-mutant competition (Methods, gray line).

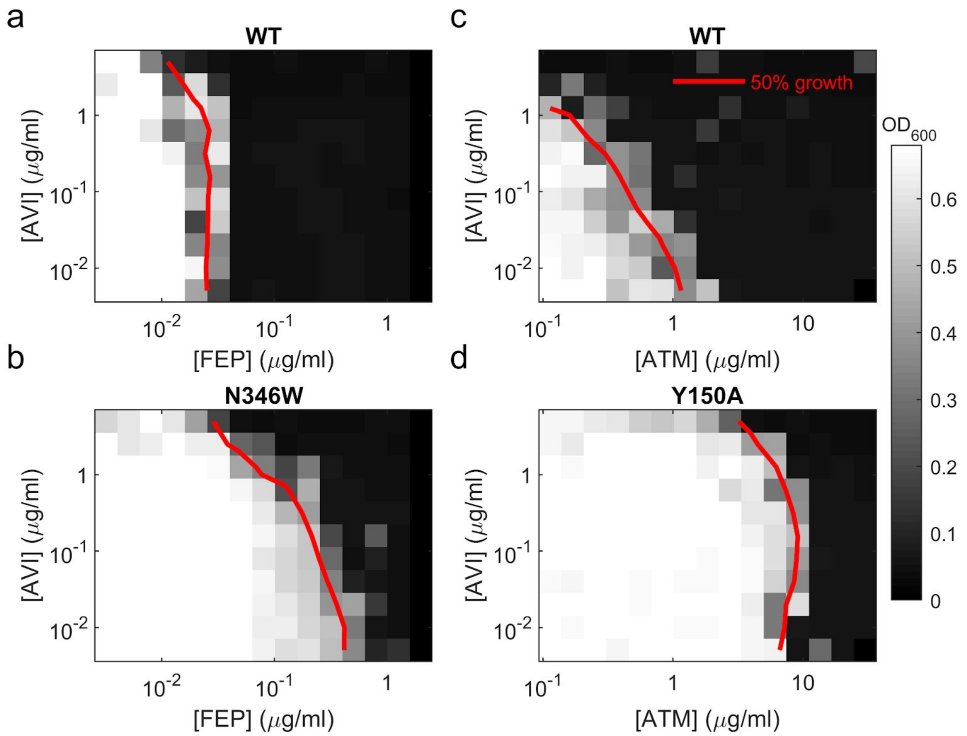

Supplementary Figure 7. Escape phenotype apear for wide range of avibactam concentration. Bacterial growth measured on 2D gradient of avibactam and cefepime (a,b) or aztreonam (c,d) presented on gray scale with the IC50 isobole, the line in concentration space where growth is inhibited by 50% (red). Escape mutant (a,c; Methods) are growing above the native MIC of the WT (b,d) even in the presence of a wide range of avibactam concentrations.

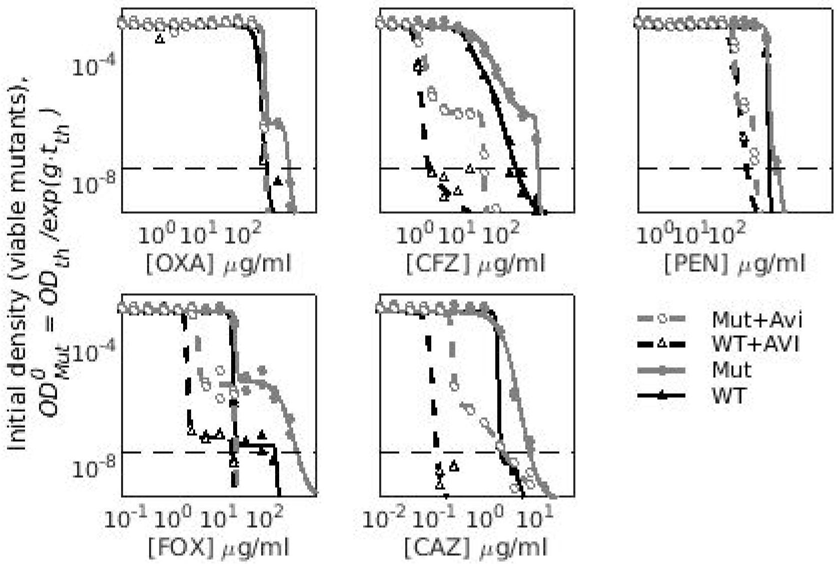

Supplementary Figure 8. The initial density of viable mutants in the additional five drugs. The initial density of viable mutants, OD^0_{Res}, is calculated from growth measurements for five beta-lactam drugs. While escape mutants exist for a narrow concentration range of cefazolin (CAZ), no escape mutations appear for the other drugs.
