# Supplementary material for: Escape mutations circumvent a tradeoff between resistance to a beta-lactam and resistance to a beta-lactamase inhibitor
Source: Nat Commun. 2020 Apr 24;11:2029. doi: 10.1038/s41467-020-15666-2 (PMC7181632; doi:10.1038/s41467-020-15666-2)
Supplement: Supplementary file 3 — Description of Additional Supplementary Files [file 41467_2020_15666_MOESM3_ESM.pdf]

Description of Additional Supplementary Files

File Name: Supplementary Data 1
Description: Oligos used in the study

File Name: Supplementary Data 2
Description: Intragenic mutations in the ampC gene alter bacterial resistance to avibactam. Drug concentrations that inhibit the growth of all mutants represented as IC50
